# Refining the Diagnosis of Growth-Related Muscle Abnormalities in Chickens Based on the Nomenclature Used to Characterise Human Myopathies

**DOI:** 10.3389/fphys.2021.745031

**Published:** 2021-11-01

**Authors:** Christophe Praud, Eva Pampouille, Elisabeth Le Bihan-Duval, Cécile Berri

**Affiliations:** ^1^INRAE, Université de Tours, BOA, Nouzilly, France; ^2^Institut Technique de l'Aviculture, Nouzilly, France

**Keywords:** white striping, wooden breast, muscle histology, muscular dystrophy, broilers

## Introduction

Although worldwide poultry meat consumption is increasing, growth-related muscle abnormalities represent a limitation to the sustainability of chicken meat production due to their significant economic consequences and unfavourable impact on product quality and social acceptability (Kuttappan et al., 2012b). Several abnormalities have already been described in modern broiler strains, but there has been a notable increase in the prevalence of white striping (WS) and wooden breast (WB) syndromes over the past decade. Briefly, the WS defect corresponds to the appearance of white streaks mainly on the *Pectoralis Major* (PM) muscle of the fillet (Kuttappan et al., 2012b), while the WB defect corresponds to a hard, bulging PM muscle with a slimy appearance and petechiae in the most severe cases (Sihvo et al., 2014). Several studies have shown that rapid growth, high slaughter weight and breast meat yield as well as limited muscle glycogen store contribute to increase the incidence of such breast muscle abnormalities (Kuttappan et al., 2013a; Abasht et al., 2016; Alnahhas et al., 2016; Kawasaki et al., 2018.

In recent years, many studies have been carried out to understand the molecular mechanisms underlying the development of breast muscle abnormalities in broiler. Muscle histological characterizations have allowed a detailed description of injuries associated with the abnormalities. Combined with high-throughput molecular studies, they have speed up the understanding of the biological processes involved in their establishment (for review, Soglia et al., 2021). In particular, these studies have shown that WS and WB muscle abnormalities share several histological features with human myopathies (Dubowitz et al., 2020) that could be used for their diagnosis (Praud et al., 2020). The purpose of this opinion paper is to highlight the importance of using current knowledge on human myopathies and existing nomenclature used by muscle pathologists to accurately interpret histological observations of chicken muscle abnormalities and thus improve their diagnosis.

## Histological Features of White Striping

The WS defect has a significant impact on breast meat composition and visual aspect of commercial broiler lines. It is first classified on the basis of macroscopic observation of the PM muscle 24 H post-mortem. Three classes are established: a normal WS0 class with no distinct white striations, a moderate WS1 class where white striations are easily recognisable, and a severe WS2 class with quite noticeable white striations parallel to the muscle fibres (Kuttappan et al., 2012b). Microscopic observations of PM muscle affected by WS describe muscle fibre degeneration, lipidosis (defined as an adipocytic infiltration), fibrosis, nuclei internalisation (Kuttappan et al., 2013b), fibre necrosis and great fibre size variability and inflammatory infiltrates (Mazzoni et al., 2015). Some fibres lost their polygonal shape to become round fibres (Sihvo et al., 2014). In addition to all these histological criteria the presence of hypercontracted fibres and frequent regenerating fibres were also described as major features of WS (Pampouille et al., 2019; Praud et al., 2020). Interestingly, we also observed that some fibres become oxidative (Praud et al., 2020) as observed in chicken muscular dystrophy (Barnard et al., 1982). The increase in macroscopic alterations is clearly related to the increased frequency of histopathologic lesions (Kuttappan et al., 2013b).

## Histological Features of Wooden Breast

The WB defect is frequently associated with WS but not always. Macroscopically, the muscle appears hard, curved, viscous and pale. Histologically, Sihvo et al. (2014) observed rare fibre splitting, sometimes necrosis and regeneration with associated fibrosis as well as perivascular inflammatory cells infiltration. Some authors have also described an accumulation of interstitial connective tissue and collagen as well as inflammatory cell infiltrates (Velleman and Clark, 2015; de Brot et al., 2016; Soglia et al., 2016). Others described vacuoles events as small vacuoles and large rimmed vacuoles (Hosotani et al., 2020). Round fibres, fibre necrosis and regeneration, hypercontracted fibres and vacuoles are most common in WB than in WS (Praud et al., 2020). Adiposis and fibrosis are most extensive in WB muscles than in WS muscles (Praud et al., 2020). And fibres exhibiting oxidative activity, usually absent in a PM muscle, were also observed to a greater extent than in WS muscles (Praud et al., 2020). Despite slow-type myosin-binding protein C is overexpressed at the mRNA level (Papah and Abasht, 2019), fibres expressing slow myosin isoforms are not observed by immunohistochemistry (Praud et al., 2020).

## WS and WB Show Histological Alterations Reminiscing Some Human Muscular Dystrophies

Since the appearance about 10 years ago of the WS and WB defects, and more recently Spaghetti Meat, the scientific community has become accustomed to gather them under the generic term of Breast Muscle Myopathies. However, referring to the terminology used for human muscular pathologies retrieved in the human phenotype ontology (HPO) database (Robinson et al., 2008), myopathy (HP:0003198) is a generic term used to define a muscle disorder without innervation or neuromuscular junction impairments with great variability in histological findings. Thus, the term myopathy encompasses all muscular dystrophies, congenital myopathies, myopathies with vacuoles, metabolic (glycogen storage-related, lipid-related, or mitochondrial), inflammatory and toxic myopathies. Muscular dystrophy (HP:0003560) defines a group of primary myopathies with a genetic basis showing progressive muscle weakness and wasting. Seventy four genes coding for muscle proteins are responsible for muscular dystrophies (Benarroch et al., 2021). The main histological features for diagnosing muscular dystrophies are the presence of necrotic, regenerating and hypercontracted muscle fibres, fibres splitting and moderate to severe extension of fibrous connective tissue (Straub et al., 2018; Dubowitz et al., 2020). Muscle fibre-switching towards slow-type and more oxidative phenotypes are also frequently observed in cases of muscular dystrophy but are not specific to these disorders as they are also present in some congenital myopathies. Inflammatory response is also a contributor of muscular dystrophies like Duchenne muscular dystrophy (DMD) (Rosenberg et al., 2015) or some limb-girdle muscular dystrophies (LGMDs) (Benveniste and Romero, 2011). Therefore, given that the histological features consistently reported in cases of WS and WB are moderate to severe extension of adipose and connective tissue, fibre necrosis and regeneration, presence of hypercontracted fibres, fibre switching, and immune cell infiltration, could be defined as muscular dystrophies rather than myopathies.

## WS and WB Are Probably Not Metabolic Myopathies

Metabolic myopathies are caused by defects in cellular energy metabolism, including the breakdown of carbohydrates and fatty acids to generate adenosine triphosphate, predominantly through mitochondrial oxidative phosphorylation (Berardo et al., 2010). In the first histological description of WS, the authors describe an adipose tissue deposition and used the term lipidosis (Kuttappan et al., 2012a), a term frequently used thereafter by other authors (Soglia et al., 2016; Baldi et al., 2018). However, in muscular diseases, the term lipidosis is associated with a large accumulation of lipids in some muscle fibres showing a vacuolar appearance but not with extracellular adipose tissue deposition (Fischer et al., 2007). Therefore, the term fatty infiltration would be more accurate to describe the adipocytic infiltration observed in WS but also WB chicken muscles, as this feature has been associated with molecular regulations related to adipose tissue differentiation, lipid transport and lipid storage (Papah et al., 2018; Pampouille et al., 2019; Praud et al., 2020). In humans, lipidosis is associated with specific metabolic myopathies classified as lipid-related disorders (Dubowitz et al., 2020). Therefore, the use of the term lipidosis to describe the ectopic adipose tissue deposition in WB muscle (Lake et al., 2019) has led authors to classify WB as a metabolic disorder, which is probably not the case. Similarly, the evidence of a metabolic shift (towards a slow type predominance) frequently observed in WB muscle (Brothers et al., 2019; Papah and Abasht, 2019; Praud et al., 2020) are not sufficient to classify WB as a metabolic disorder, as this phenomenon like the presence of ectopic adipose tissue can be observed in several types of muscular dystrophies such as DMD and some LGMDs (Dubowitz et al., 2020). Moreover, several features observed in WS or WB muscles, such as fibrotic and adipose tissue deposition, hypercontracted fibres, or fibre-type switching, are not typically observed in the three major categories of human metabolic myopathies, namely glycogen storage diseases, fatty acid oxidation defects, and mitochondrial disorders (Dubowitz et al., 2020).

## Regenerative Processes are Mainly Responsible of Fibre Size Alteration

Another typical feature of WS and WB diseases is the wide variation in muscle fibre size. In particular, there is an increased number of small fibres (Mazzoni et al., 2015) sometimes described as small calibre fibres (Hosotani et al., 2020) and a description of split fibres (Sihvo et al., 2014; Hosotani et al., 2020). Small calibre fibres traditionally refer to atrophic fibres, while split fibres refer to the longitudinal halving of the entire fibre (Dubowitz et al., 2020). Accordingly, the small-calibre fibres described in Hosotani et al. (2020) are most likely regenerating fibres since they contained central nuclei and appear more basophil than the others. The split fibres often described in WS and WB muscles are also likely regenerating fibres consecutive to a segmental necrosis as they expressed the ventricular myosin heavy chain isoform, MYH15 (Praud et al., 2020), previously described as a marker of the regeneration process (Camoretti-Mercado et al., 1993). Moreover, observation of longitudinal sections of muscle fibres (Papah et al., 2018) revealed signs of segmental necrosis. Large size fibres are also frequently observed in WS and WB muscles. They are described as round and hypertrophic (Wang et al., 2020) or as hypercontracted fibres (Praud et al., 2020) and are known to be mainly associated to dystrophic conditions in humans (Dubowitz et al., 2020).

## The Vacuolization of the Fibres Could Be Due to An Altered Regulation of the Autophagic Process

Hosotani et al. (2020) has recently described both small and large rimmed vacuoles in WB muscle fibres. However, it seems that the large vacuoles observed do not exactly have the characteristics of rimmed vacuoles as classically defined by muscle pathologists (Dubowitz et al., 2020). In abnormal muscle fibre morphology section (HP:0004303) of HPO database, rimmed vacuoles (HP:0003805) are defined as clear vacuoles with a densely blue rim on hematoxylin and eosin (HE) muscle cross-sections that are often associated with cytoplasmic inclusions. The blue rim is usually observed as a basophilic granularity at the vacuole periphery after stained in red with the Gomori's trichrome staining (Fukuhara et al., 1980). However, neither the blue rim on HE sections nor the red staining after Gomori's Trichrome is present around vacuoles in chicken muscles affected by WB (Hosotani et al., 2020; Praud et al., 2020). In ultrastructural observations, rimmed vacuoles show some multi-laminated membranous structure and finely granular material without membrane at the rim of the vacuole (Fukuhara et al., 1980). However, vacuoles observed in chicken WB muscles are surrounded by a dotted line and do not have a multi-laminated membranous structure (Hosotani et al., 2020). Hosotani et al. (2020) also suggested that the vacuoles they observe are autophagic vacuoles like those observed in Pompe and Danon diseases, two primary lysosomal glycogen storage myopathies. However, in these diseases the vacuoles are stained for glycogen after a periodic acid Schiff staining, which has yet to be demonstrated in chickens. The presence of hypertrophic and regenerating fibres, as well as areas of necrosis and inflammatory infiltrates frequently described in the WS and WB muscles, are usually not associated with Danon and Pompe disease, which does not argue for a similarity between chicken muscle pathologies and these two types of myopathies. However, autophagic vacuoles have been observed in a wide range of myopathic disorders, including inclusion body myositis and many muscular dystrophies, implying autophagy dysregulation (Castets et al., 2016). Therefore, further studies are needed to better understand the role that autophagic processes play in WS and WB pathogenesis.

## Conclusion

In recent years, a considerable research effort has been made to understand the origin of growth-related muscle abnormalities in chickens. Thanks to molecular and histological analyses, it has been possible to identify a large number of biological deregulations, but the causes, especially genetic, still remain to be elucidated. Through this opinion paper, we underline the importance of using the large amount of knowledge acquired on human myopathies, especially dystrophies, to refine the diagnosis and to progress in the search for solutions to reduce their incidence.

## Author Contributions

All authors listed have made a substantial, direct and intellectual contribution to the work, and approved it for publication.

## Conflict of Interest

The authors declare that the research was conducted in the absence of any commercial or financial relationships that could be construed as a potential conflict of interest.

## Publisher's Note

All claims expressed in this article are solely those of the authors and do not necessarily represent those of their affiliated organizations, or those of the publisher, the editors and the reviewers. Any product that may be evaluated in this article, or claim that may be made by its manufacturer, is not guaranteed or endorsed by the publisher.
